# Development of a Scalable Testbed for Mobile Olfaction Verification

**DOI:** 10.3390/s151229834

**Published:** 2015-12-09

**Authors:** Syed Muhammad Mamduh Syed Zakaria, Retnam Visvanathan, Kamarulzaman Kamarudin, Ahmad Shakaff Ali Yeon, Ali Yeon Md. Shakaff, Ammar Zakaria, Latifah Munirah Kamarudin

**Affiliations:** 1 Centre of Excellence for Advanced Sensor Technology (CEASTech), Universiti Malaysia Perlis, Pusat Pengajian Jejawi II, Taman Muhibbah, Arau, Perlis 02600, Malaysia; retnamvr7@gmail.com (R.V.); arul.unimap@gmail.com (K.K.); ahmadshakaff@gmail.com (A.S.A.Y.); aliyeon@unimap.edu.my (A.Y.M.S.); ammarzakaria@unimap.edu.my (A.Z.); latifahmunirah@unimap.edu.my (L.M.K.); 2 School of Mechatronic Engineering, Universiti Malaysia Perlis, Pauh Putra Campus, Arau, Perlis 02600, Malaysia; 3 School of Microelectronic Engineering, Universiti Malaysia Perlis, Pauh Putra Campus, Arau, Perlis 02600, Malaysia; 4 School of Computer Engineering, Universiti Malaysia Perlis, Pauh Putra Campus, Arau, Perlis 02600, Malaysia

**Keywords:** mobile olfaction, gas sensors, mobile robots, sensor array, experiment verification

## Abstract

The lack of information on ground truth gas dispersion and experiment verification information has impeded the development of mobile olfaction systems, especially for real-world conditions. In this paper, an integrated testbed for mobile gas sensing experiments is presented. The integrated 3 m × 6 m testbed was built to provide real-time ground truth information for mobile olfaction system development. The testbed consists of a 72-gas-sensor array, namely Large Gas Sensor Array (LGSA), a localization system based on cameras and a wireless communication backbone for robot communication and integration into the testbed system. Furthermore, the data collected from the testbed may be streamed into a simulation environment to expedite development. Calibration results using ethanol have shown that using a large number of gas sensor in the LGSA is feasible and can produce coherent signals when exposed to the same concentrations. The results have shown that the testbed was able to capture the time varying characteristics and the variability of gas plume in a 2 h experiment thus providing time dependent ground truth concentration maps. The authors have demonstrated the ability of the mobile olfaction testbed to monitor, verify and thus, provide insight to gas distribution mapping experiment.

## 1. Introduction

Implementing gas sensing on real robots is a major research focus in the gas sensing field due to the numerous potential applications that mobile gas sensing systems may contribute to Labour intensive and hazardous applications such as detecting gas leakage in mines and factories, finding concealed landmines and locating illegal substances may benefit from advances in this research field [1]. Such applications may no longer depend on trained animals or put humans in harm’s way in the future. Collaborative robot research may also benefit from the findings in this field as chemical trails or markings may be used in the interactions between different robots [2]. Current research trends in mobile olfaction is structured around tasks such as gas distribution mapping [3], gas trail tracking [2], gas plume tracking [4] and gas source declaration [5].

The development of mobile olfaction strategies in the previously said tasks requires an understanding of how gas disperses in the environment under turbulent airflow. Marques commented that validation of experimental data is the most common weakness in mobile olfaction experiments [6]. Due to the difficulties in creating a fully controlled test environment, most researchers use simulations before verifying it with an actual run. This requires creating a mathematical model [7] or collecting the average gas dispersion in the test area and using the dataset in the simulation as described previously. A mathematical model of the gas dispersion requires high processing power; although simplified models are available, it may not describe gas dispersion and gas sensor characteristics as accurately. To overcome this deficiency, the ability to gauge the instantaneous gas dispersion at any time during the experiment to facilitate understanding of gas dispersion and the tested system is needed. This ability would allow verifications of experimental data to be done with accuracy.

Presently, most researchers create a gas dispersion model to simulate and aid the development mobile olfaction strategies [4,8,9,10]. The different models for gas dispersion that has been used for simulation are such as averaged gas dispersion [11], filamentous dispersion [7,12], CFD [13] and Gaussian distribution [14]. These models, although are a good representation of the odor dispersion, it does not fully capture the unpredictable nature of gas dispersion. The best possible simulation would use streamed data collected from an actual test environment, however this has yet to be done to date.

Ishida *et al.* used real measurements of gas concentrations at multiple points in the clean room testbed using a fixed grid of MOX gas sensors [15] to create a dispersion map. Wind speed and direction measurements were also made to verify the air movement in the test area. Others have made a series of single point gas concentration measurements to estimate the gas dispersion [16]. Instead of averaged gas sensor readings, the maximum reading at each point was used. Conversely, Pyk *et al.* used the time averaged gas sensor reading to create a gas dispersion profile. Using a single sensor, 2 min gas concentration measurement were made at multiple points in the testbed to produce a time-averaged map [17]. The average wind speed was calculated by measuring the wind speed at the outlet of the wind tunnel and reconstructing wind speed and air volume. Maps were also created using concentration and wind direction measured by moving robots. With the advancement of mobile olfaction, some researchers use robots to aid gas concentration mapping. For example, a series of single point measurements were made at pre-defined grid points using a mobile robot [18]. Gas readings were measured with an electronic nose while wind speed readings were made using an anemometer. Similarly, a series of single point measurements made by 6 robots moving randomly in the testbed was also used to record “odor hits” in the test area [19]. The definition of odor hits is that when the gas sensor response exceeds a set threshold. Similar to Pyk, wind speed and direction readings were also taken by the robots. A Gas Sensor Network was also used to map the gas dispersion in a 3 m by 4 m test area [20]. However, only an instantaneous dispersion map was used as distribution profile in their simulations. Quantitative study on gas dispersion was reported using a set of gas sensor arrays [21,22]. The gas sensor array is composed of eight gas sensors to detect different types of analytes and monitor its concentration in the environment. A normalized map of the gas dispersion was presented in the initial work and then gas mixture concentration was estimated using gas chromatography-mass spectrometry (GC-MS).

These methods are only provide time-averaged or instantaneous concentration maps; the uncontrollable, time-varying and unpredictable nature of gas dispersion is lost and may not be properly described. This becomes a challenge in mobile olfaction testing and as unexpected peaks and troughs in gas dispersion cause the tested system to react erratically [23]. As a result, different experiment runs may produce varying results as even though the average gas distribution is the similar, the instantaneous gas plume formed during the experiment is different. Consequently, comparison between different mobile olfaction strategies may become inconclusive due to the different test conditions.

Furthermore, real world experiment runs also tend to consume a lot of time—each run takes at least 10 min [24,25,26,27,28]. Not only the experiments themselves take considerable times; post-run conditioning in between experiment runs (resetting gas source and clearing up the air in test environment) requires a long wait [6]. In addition, extended experiment times releases more gas into the test environment, which may saturate the environment with accumulated gas. This, in turn, increases the wait time between consecutive experiment runs. In our experience, the wait time can be up to 30 min. Accumulated gas in the experiment environment, if not monitored properly, may skew results of subsequent experiments.

To recapitulate, the mobile olfaction experiments are not straight forward as there are many uncontrollable variables that come into play and affect experiment results. The biggest source of uncertainty in experiments is the gas dispersion in the test area making verification of experimental data impossible without specialized tools. This paper documents the development of an integrated system for mobile robot olfaction experiments which may be used to create ground truth information to verify experimental results. The designed testbed design also tries to overcome the shortcomings in gas plume data acquisition and describe how the system can facilitate deeper understanding mobile robot olfaction navigation algorithms. The authors will demonstrate the use of the testbed with a simple gas distribution mapping experiment presented in the Results and Discussions Section.

## 2. Mobile Olfaction Testbed Design

**Figure 1:**
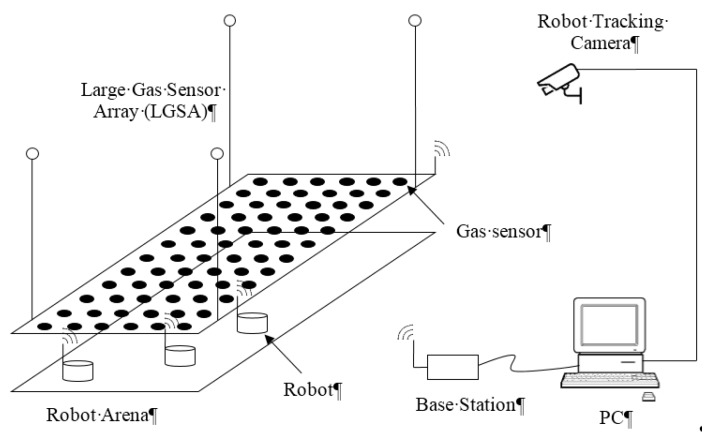
Overview of the testbed system.

The system presented in this paper integrates various subsystems of mobile olfaction data collection, verification and testing infrastructure. Each subsystem was designed to be able to function independently which allows different modes of uses for different types of experiments. The main subsystems of the testbed are shown in Figure 1.

### 2.1. Large Gas Sensor Array (LGSA)

The Large Gas Sensor Array (LGSA) is designed to monitor and log the gas dispersion in a 3 m-by-6 m robot testbed. The main aim of this subsystem is to observe the gas dispersion at different heights and its temporal changes. Recorded gas dispersion data may be fed into a simulation, verify experiments and enhance understanding of mobile robot olfaction behavior. To achieve these objectives, an array of gas sensors distributed in the testbed is proposed.

**Figure 2:**
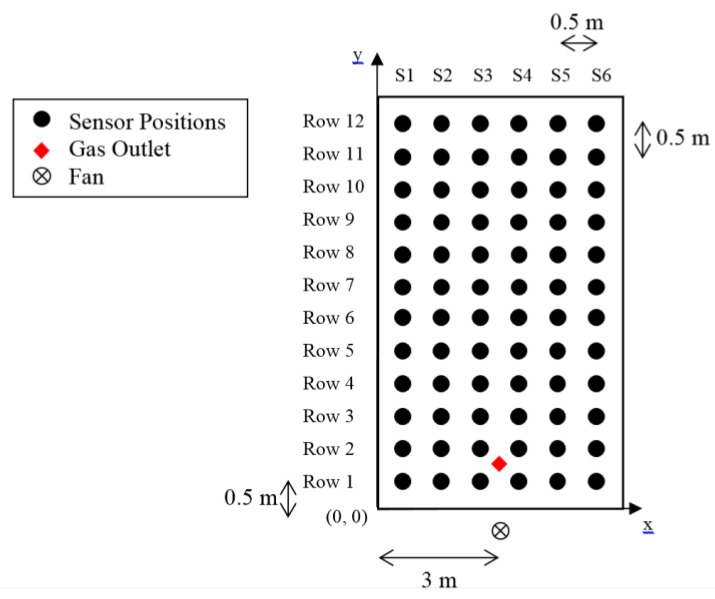
Sensor positions in the array.

The system consists of an array of 72 MOX gas sensors, which are arranged in a 12-by-6 grid. The sensors are separated with a distance of 0.5 m from each other as depicted in Figure 2. Each of the 12 rows is monitored via a WSN transmitter which samples the sensor reading and transmits the collected data to the base station.

**Figure 3:**
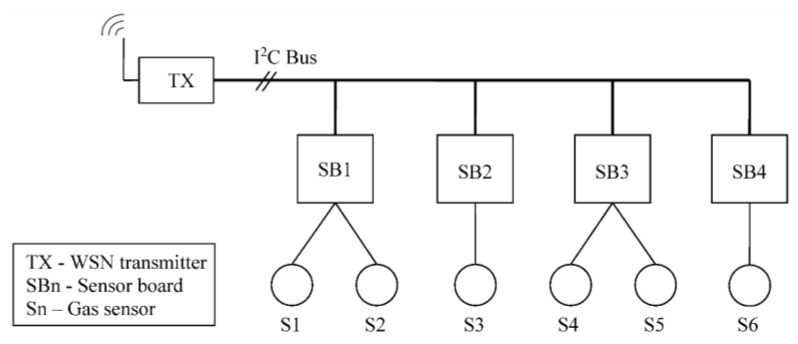
Transmitter-sensor interfacing.

Sensor sampling is done using a specifically designed board based on Microchip’s MCP3427 delta-sigma ADC IC. These boards are placed as close as possible and connected via shielded cable to the sensors to reduce noise. Each 12-bit sensor board can be connected to up to two gas sensors and has a conversion rate of 240 samples per seconds. In each row, a total of four sensor boards used to measure readings from the six gas sensors. Measurements are requested by the WSN transmitter from the sensor boards every 1 s via I2C serial communication protocol. A diagram describing the interaction between the sensor boards and the wireless transmitter is shown in Figure 3.

**Figure 4:**
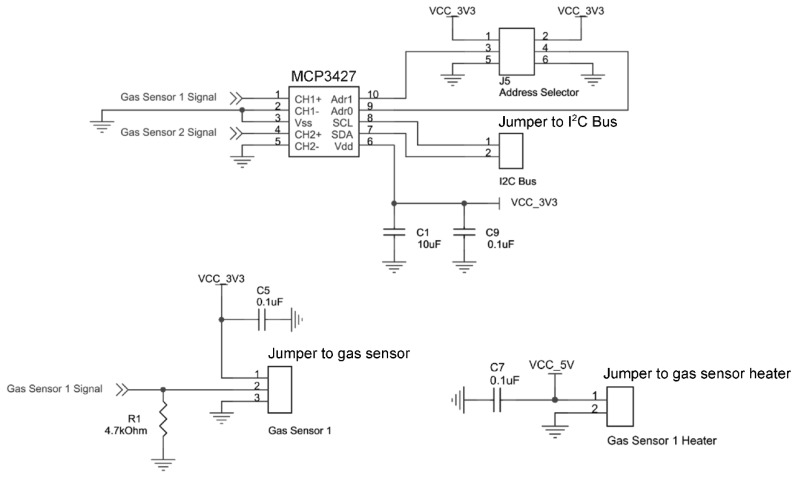
Gas sensor interface circuit and schematics of sensor board.

In this paper, all experiments were conducted with ethanol. Consequently, all 72 gas sensors used in this system are TGS2600 from Figaro as it is sensitive to ethanol; although it is possible to connect other types of sensors to the system. A typical voltage divider circuit is used to detect resistance changes in the gas sensor. Figure 4 shows the sensor board schematic design. A low resistance of 4.7% ± 1% kΩ is used as load resistor, R_L_, to enhance sensitivity at lower gas concentrations. The heater is supplied with *V_H_* = 5 V while the sensor side is supplied with *V_C_* = 3.3 V. Lower voltage was used on the sensor side to cater to the needs of the system without the need for more interface circuitry; the wireless transmitter communicates at 3.3 V level thus requiring the sensor board to communicate and operate at the same voltage.

The metal oxide sensor is affected by environmental changes such as humidity and temperature especially in open sampling systems. As the gas sensor is sampling in a partially controlled room environment, it can be assumed that all gas sensors are subjected to the same conditions. Consequently, the normal ratio of the resistance change with respect to the baseline is taken as the sensor signal, *i*; with range [0, 1].

(1) $$s=\frac{\Delta \text{R }}{R0}=\frac{R_{0}-R_{S}}{R_{0}}=1-\frac{R_{S}}{R_{0}}$$

where *R_0_* is the baseline resistance and *R_s_* is the sensor response. The sensor reading before ethanol is released is taken to be the baseline reading, *R_0_*.

### 2.2. Wireless Data Network

Wireless Sensor Network (WSN) transceivers were used to create a communication backbone for the different components of the testbed. This approach was adopted to minimize wiring effort of the LGSA system and to ensure deployment flexibility and scalability of other different subsystems. The wireless communication is based on commercially available WSN transceiver (MEMSIC’s XM2110CA) with customized firmware. The transceivers use the Atmel RF230 IEEE 802.15.4-2003 compliant radio integrated with an Atmega1281 microcontroller and has a maximum data rate of 250 kbit/s. The MEMSIC WSN nodes are also capable of forming a mesh network and data packet hopping which is useful for future widespread testing and deployment. In this system, communications between transceiver and the base station are asynchronous. However, in order to reduce the collision and packet drops in the LGSA, a delay was implemented during the transmitter start-up to stagger the transmission time is described as follows:

(2) $$Delay=\frac{1}{12}(n-1)$$

where *n* = 1 ... 12 denote the rows of the LGSA.

**Figure 5:**
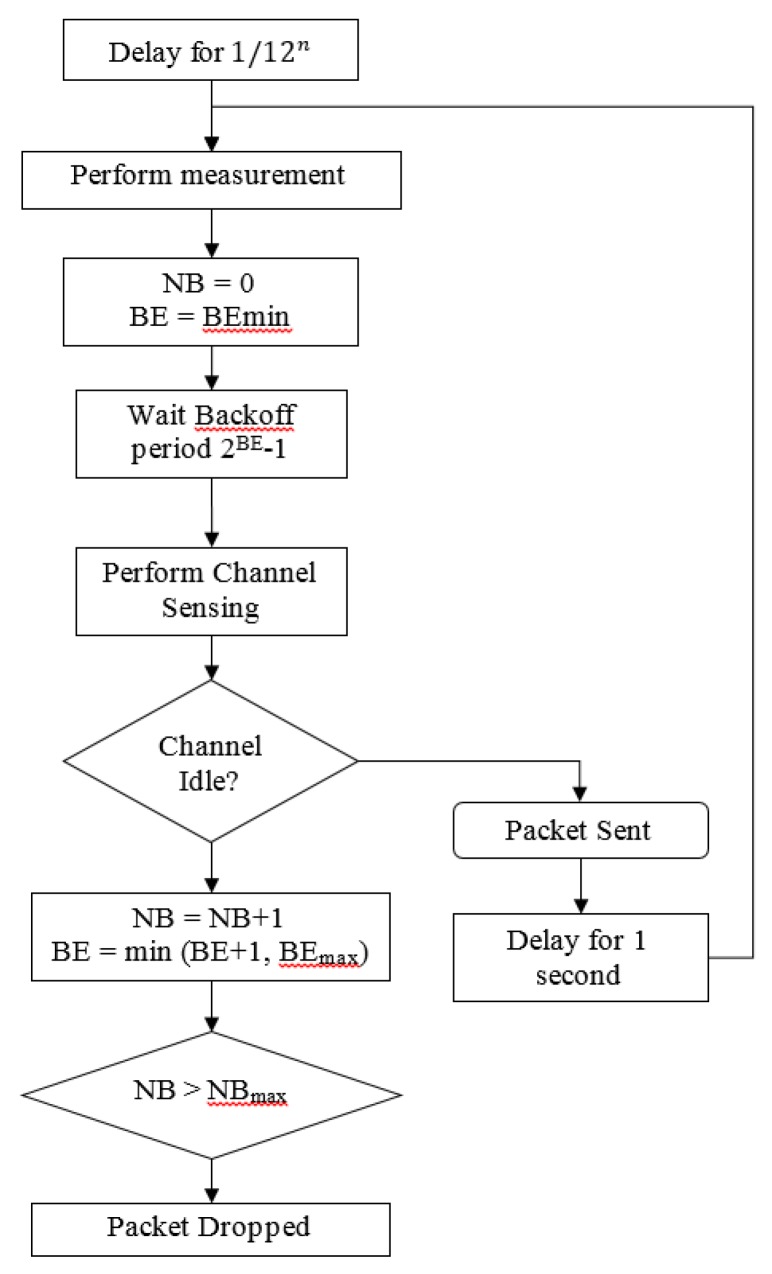
WSN transmitting flowchart; where BE is backoff exponent and NB is the number of successive backoffs before current transmission.

At the beginning of every data transmission, the transceiver executes the CSMA-CA to assess the channel. If a clear channel is detected, the radio transceiver proceeds to transmit the frame. After frame transmission, the radio transceiver switches into receive mode to wait for an ACK. If no valid ACK is received or a timeout (after 864 µs) occurred, the transceiver retries the entire transaction, including CSMA-CA execution. These processes are repeated until the frame has been acknowledged or the maximum number of retransmissions has been reached. In this case, the maximum number of retransmission is set to 7. Once transmission is successful, the transceiver moves to an idle state until the next cycle begins. The interval between two data transmissions is denoted as cycle. The transmission flowchart summarizing the whole process is shown in Figure 5.

In the testbed system, a single base station acts as a data sink for data packets from the robots and the LGSA. Data packets received from the gas sensors and the robots are forwarded via USB to a PC to be displayed and recorded. The data from the robots may also be retransmitted to specific robots depending on the operating needs. Although it is possible for robots to send and receive messages to each other without passing data through the base station, however, for data collection and battery charge conservation purposes, data are always transmitted to the base station. Different functional options are also intentionally designed into the robot’s communication system for future system deployment and scalability. The 12 nodes in the LGSA act as transmitters; sending sensor readings from six gas sensors each to the base station. All collected data and time of receipt of each packet are recorded in a dated file and displayed in a graphical user interface for real-time monitoring of experiments. As all data are transmitted wirelessly, the monitoring station may be placed farther, thus avoiding human caused interference to experiments.

### 2.3. Design for Robot Integration with Testbed

**Figure 6:**
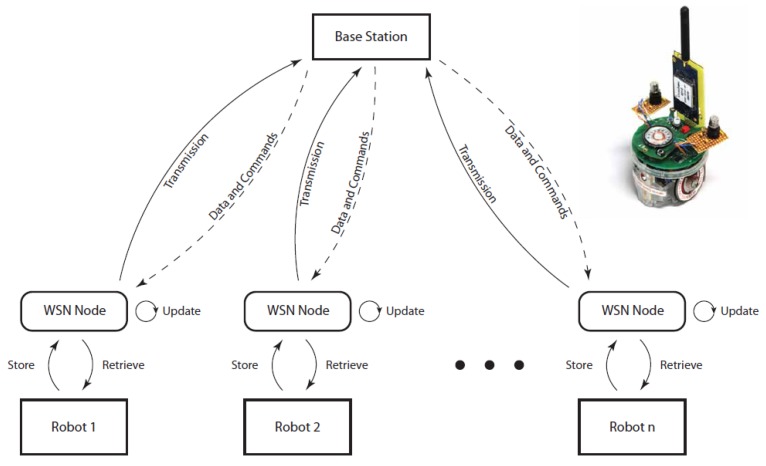
Overview of Robot interactions via WSN and an example of a robot with a WSN transceiver.

A WSN node is connected to each robot to allow it to communicate with other robots and the base station (seen in Figure 6). Communication between the WSN node and the robot is based on I2C. The WSN node stores data received from the base and other robots and only passes the data to the robot if requested by the robot. Assigning the communication and data maintenance to the WSN node reduces the load on the robot’s controller. The robot sends its current status and sensor readings to update the data in the connected WSN node. The WSN node will then in its own time transmit the data to the base to be monitored and recorded or directly to other robots. It is also possible for the robot to request its status and sensor readings immediately if needed. The WSN manages all data packets and keeps track on updates its data to the latest data packet. Figure 6 represents the data movement between the robot, the WSN node, the base station, and other robots.

### 2.4. Robot Tracking System

A robot tracking system was developed to verify robot odometry results and to simplify robot integration into the testbed. An accurate robot positioning system is important when recording experimental results. Although robots may be able to perform odometry and transmit its position to the base station, the error in the robot’s odometry may affect the accuracy of the experiment results. Furthermore, robot integration into the system may be simplified by reducing the need to implement odometry or other position tracking methods on the robot themselves.

**Figure 7:**
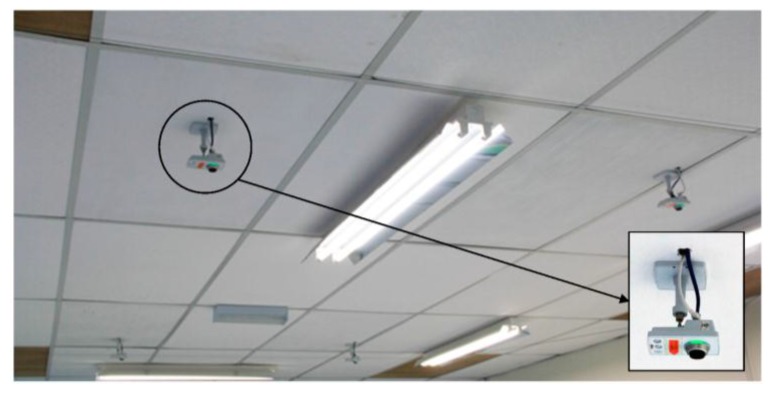
Robot tracking cameras over the test area.

Bird’s eye view is a common method used to monitor and track the trajectory of moving robots. This configuration was used to avoid robots of different heights to disrupt the tracking of another robot. The system consists of 4 Axis M1034-W network cameras, mounted on the ceiling which covers the test area below as shown in Figure 7. The camera provides motion JPEG with a maximum resolution of 1280 × 800 pixels and frame rate of 30 fps.

Robot locations are tracked using pattern matching algorithm. Pattern templates were created and used as reference pattern. Then, the algorithm searches for the trained pattern and determines its position and orientation within the captured image. Since the algorithm matches pattern based on its geometric feature and intensity, multiple patterns can be matched within a single image. A total of twenty patterns have been trained. These patterns are then placed on robots for localization. The developed system is able to track and localize multiple robots for every 500 ms with a maximum error of ±1 cm.

### 2.5. Monitoring Software Design

**Figure 8:**
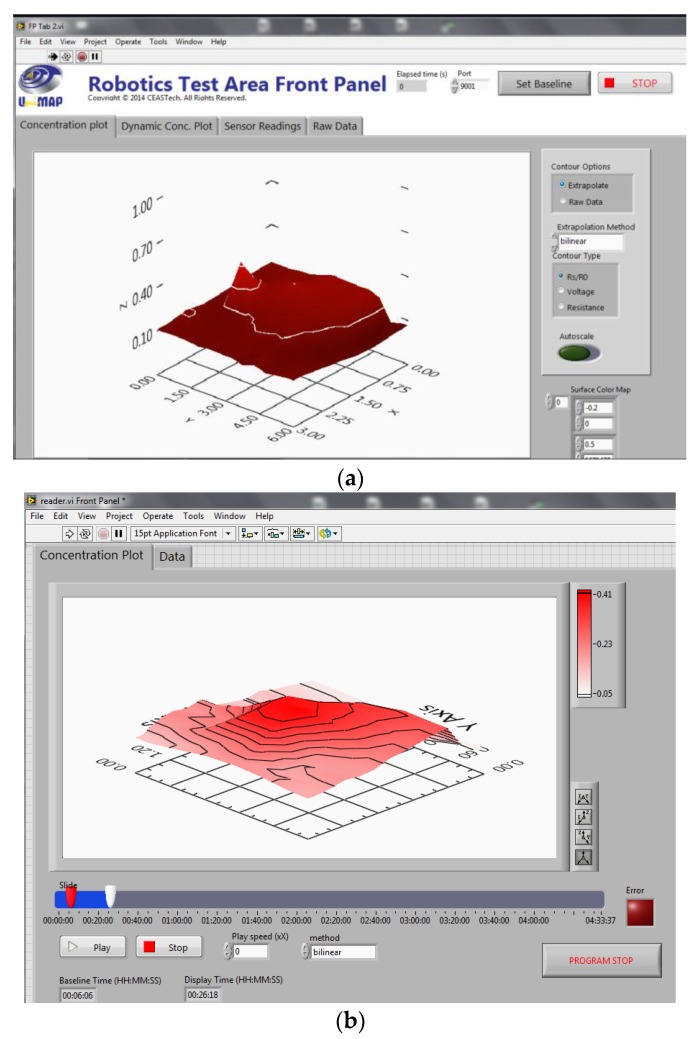
Screenshot of the (**a**) Large Gas Sensor Array (LGSA) monitoring interface and the (**b**) LGSA player.

User interfaces were developed to view the collected sensor and robot data in real-time or to replay the collected data. All software was developed using LabVIEW. The user interface for the LGSA, the LGSA player are shown in Figure 8. The LGSA interface gives information to the user about the current status of the system, from the sensors’ current voltage, resistance and signal readings for each row. A surface plot is also included to visualize the estimated gas dispersion in the testbed. The LGSA player allows user to review the collected data and quickly grasp the movement of the gas in the testbed. Users can set the speed of the replay manually to expedite analysis. The robot tracking system displays data collected from the robot and its real position in the testbed.

### 2.6. Simulation Environment

Webots, a development environment software, was used to investigate the performance of robots performing mobile olfaction tasks. The simulation environment allows the emulation of various types of sensors, including proximity sensors, LIDAR, gas sensors and actuators such as motors and servos. Physical properties of objects in the simulation environment can also be set to emulate its behavior in the real world. Gas sensors and odor plume have been emulated in the simulation environment. In this research, a virtual test area was built based on the actual testbed.

**Figure 9:**
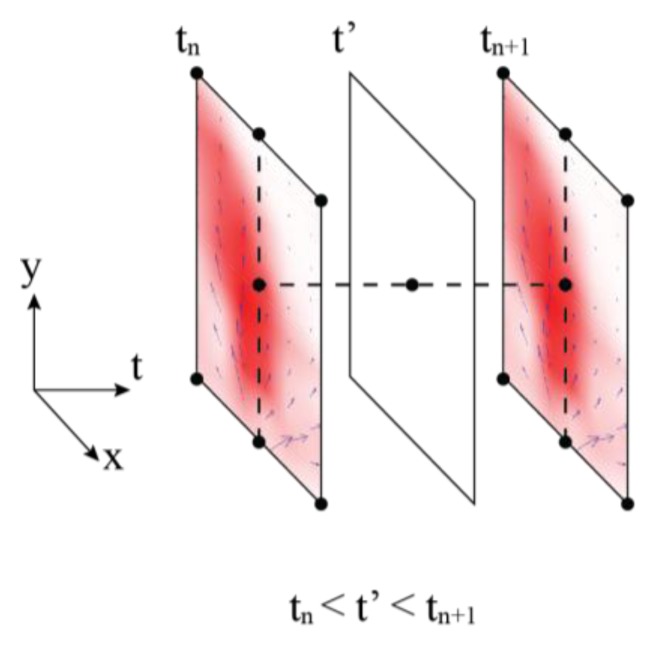
Visualization of linear interpolation between data streams.

The collected data from the LGSA was streamed into a simulation environment to provide a time dependent gas dispersion map. The data stream was interpolated to create gas concentration maps for every second of the collected data. However, simulation time, *t’* often do not coincide with the data stream time, *t_n_*. Thus, the gas concentration was also interpolated according to the simulation time and data stream time. A visualization of the linear interpolation is depicted in Figure 9.

The averaged airflow in the testbed was loaded into the simulation to simulate airflow which can be measured by a virtual anemometer. The airflow measurements are interpolated between the sample points similar to the gas dispersion map. Previous researches has proposed that airflow variations can be described as Gaussian distribution. This research in the following section, will provide data that demonstrate this behavior. Therefore, the simulation varies the averaged airflow readings such that the variations are normally distributed.

## 3. Results and Discussion

In this section, we first evaluate the feasibility of using multiple gas sensors to create instantaneous gas distribution map. The airflow variability and gas dispersion in the defined testbed is also presented to demonstrate the ability of the system to detect the unpredictability of the gas dispersion. In this section, the unpredictability of the gas plume and the lost temporal information in the time-averaged gas distribution map were also demonstrated. Additionally, the testbed system was also used to gauge and verify the gas distribution performance of two robot gas mapping method by comparing it to the data collected from the LGSA.

### 3.1. Gas Sensor Calibration and Analysis

There are a total of 72 gas sensors with the same production lot number used in the Large Gas Sensor Array (LGSA) system. Due to the large number of gas sensors in the system, the validity of the data collected depends on the accuracy and similarity in readings of different sensors when exposed with the same gas concentrations. A documented system that use multiple sensors of the same type scale the sensor readings based on a single calibration run [29]. To verify that the same readings may be produced by different sensors and that the sensor array can coherently differentiate different concentrations, 5 randomly picked sensors were put on calibration runs. This experiment mainly aims to establish the similarity of readings from different sensors when exposed to the same gas concentrations.

The setup of the calibration system was previously presented by Hawari *et al.* [30]. In this experiment, the gas sensor is exposed to different concentrations of ethanol—40, 60, 80, 100 and 120 ppm. The different ethanol gas concentrations were produced by bubbling compressed air in a 5% solution of ethanol in water at 30 °C to produce saturated ethanol gas. Then, by mixing the saturated ethanol gas with compressed air, different gas concentrations can be reliably produced. After initial purge for 300 s, the gas sensor is exposed to increasing concentrations of ethanol gas for 240 s. After each concentration, the gas sensor chamber is purged with compressed air for 360 s to allow time for the gas sensor to return to baseline [31].

**Figure 10:**
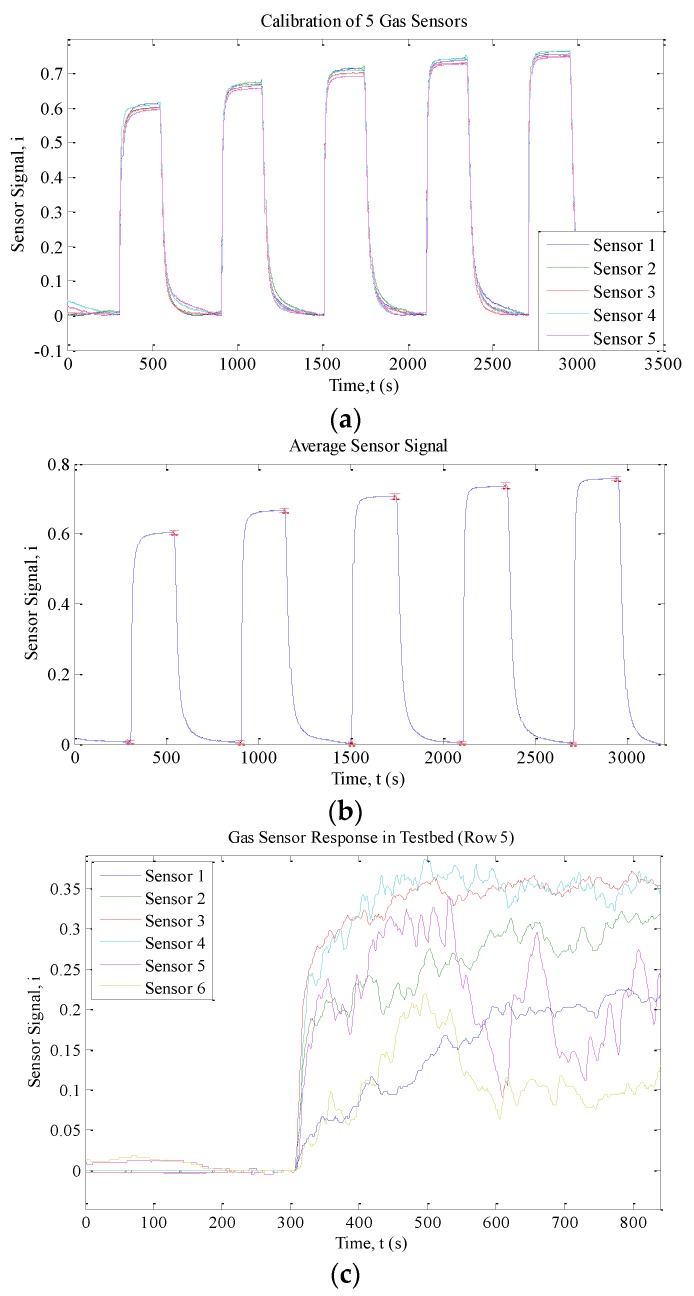
(**a**) Response of five gas sensors; (**b**) the average response; and (**c**) the response of gas sensors in the testbed.

Experiments were run in standard ambient temperature and pressure with relative humidity ranging from 50% to 65%. The sensor response under different calibration profiles is depicted in Figure 10. Table 1 summarizes the steady state readings of all sensors for the three calibration profiles.

In general, all five sensors have the same response when exposed to the same ethanol concentration. The steady state values of all five sensors are similar with small variances and standard deviations as presented in Table 1. The data were tested with ANOVA to verify that the different concentration levels can be distinguished. From Table 2, we can conclude that the steady states of the five sensors are similar and that the sensors can differentiate between different concentrations. Hence the gas sensor array operating with 72 sensors is expected to be able to produce accurate readings provided that all sensors used are from the same production lot.

**Table 1:** Summary of gas sensor calibration results.

Sensor	20 ppm	40 ppm	60 ppm	80 ppm	100 ppm
Sensor 1	0.6134	0.6677	0.7102	0.7386	0.7558
Sensor 2	0.6013	0.6728	0.7150	0.7447	0.7615
Sensor 3	0.6018	0.6638	0.7017	0.7303	0.7511
Sensor 4	0.6086	0.6698	0.7108	0.7418	0.7631
Sensor 5	0.5957	0.6575	0.6914	0.7265	0.7487
Mean	0.6042	0.6663	0.7058	0.7364	0.7560
Variance	4.7603 × 10^−5^	4.7603 × 10^−5^	4.7603 × 10^−5^	4.7603 × 10^−5^	4.7603 × 10^−5^
Std Dev	0.0061	0.0053	0.0084	0.0069	0.0056

**Table 2:** Summary of gas sensor calibration results.

Source of Variation	*SS*	*df*	*MS*	*F*	*p-Value*	*F crit*
Between Groups	0.073109	4	0.018277	338.5219	4.63 × 10^−18^	2.866081
Within Groups	0.00108	20	5.4 × 10^−5^			
Total	0.074189	24				

### 3.2. Gas Mapping Using LGSA

**Figure 11:**
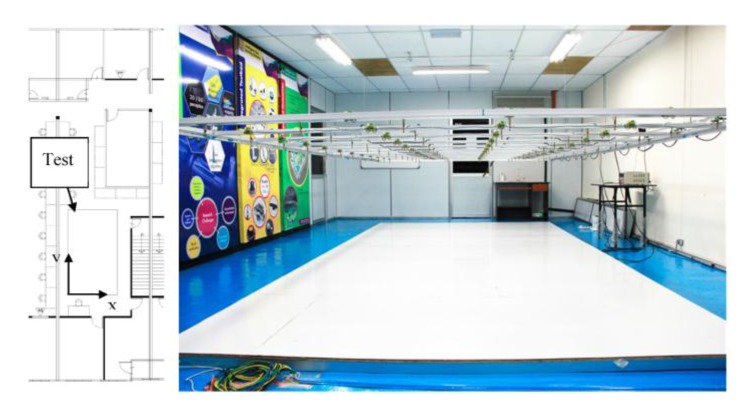
The layout of the laboratory and picture of the test area.

Real time monitoring and data storage of mobile olfaction experiments is made possible with the system proposed in this paper. As it was developed separately from the other components, the LGSA can be run independently. The gas dispersion in the test bed was recorded using LGSA to study the characteristics of gas dispersion in this specific environment. The experiment area and its layout are shown in Figure 11.

**Figure 12:**
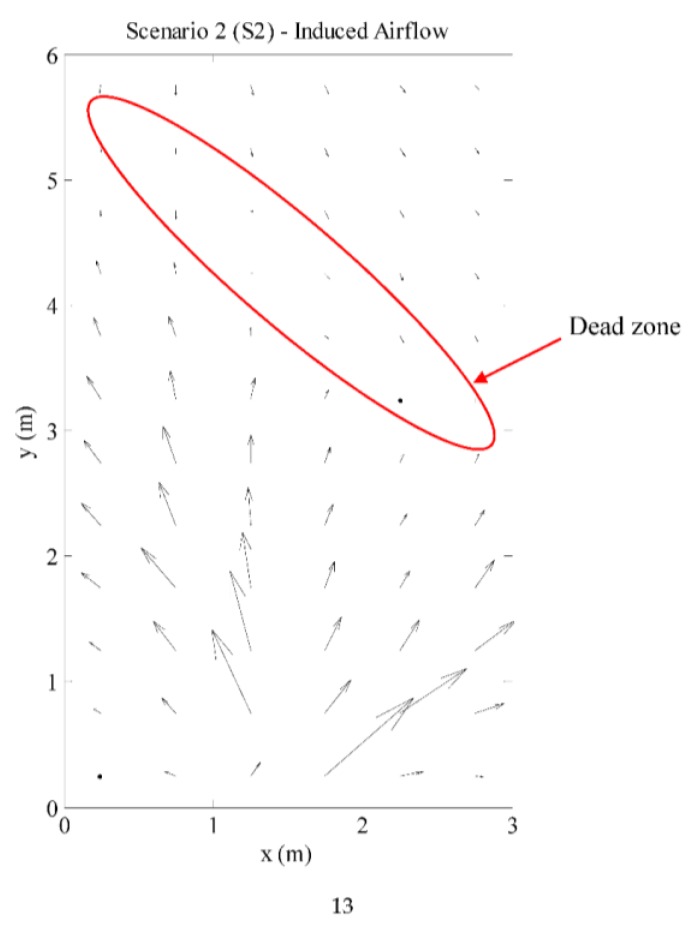
Average airflow in testbed—fan turned on.

Ethanol is introduced into the environment using a bubbler filled with 20% ethanol solution in water; with the outlet placed between Row 1 and Row 2 with coordinates (3, 0.5), marked in Figure 2. A bladeless fan (Imaha model HTWF Y-12) was used to induce air flow when needed. The area was closed off from any human activities during all experiments. The produced airflow in the testbed is presented in the following section in Figure 12.

The LGSA was lowered to a height of 0.15 m from the testbed platform for all experiments. The air movement in the testbed at each sensor position was recorded with FTTech’s LM602 anemometer. The anemometer has an air speed resolution of 0.001 m/s and angle resolution of 0.1°. Each point was sampled for 5 min at five samples per seconds. Then the gas dispersion experiment is conducted and all gas sensor readings were recorded. The experiment was run initially with 10 min without the wind source or ethanol turned on. Then, the wind source was turned on for 5 min before a 2 h continuous release of ethanol plume into the testbed. After 2 h, the bubbler was turned off to record how the air in the testbed clears up after the experiment. To ensure that the gas sensor does not drift, the sensors were warmed up for more than a week before the experiments were run.

This section will describe and discuss the data collected from the LGSA from the experiment runs. The authors acknowledge that three-dimension air movement affects the dispersion of gas in the testbed; however this paper will only consider gas dispersion along a 2-D plane pertaining to 2-D mobile olfaction strategies. The 2-D information provided by the system is adequate for the development of 2-D mobile olfaction strategies as the robot would only traverse in a 2-D space.

#### 3.2.1. Airflow Produced in the Testbed

The dispersion of odor is heavily influenced by the advection and diffusion of the gas the carrying fluid. Therefore, the air movement in the testbed is measured and studied first to understand the gas plume structure produced. The recorded air movement in the testbed is shown in Figure 12; however, the natural air movement is prevalent downwind (at the top of Figure 12). As there is induced airflow, the average wind speed is on average = 41.0 cm/s ± 41.5 cm/s with airflow speed ranging from 9.2 cm/s ± 5.3 cm/s to 214.8 cm/s ± 10.8 cm/s. There are also “dead zones”—areas with relatively low airflow, where the induced airflow meets with the natural airflow.

**Figure 13:**
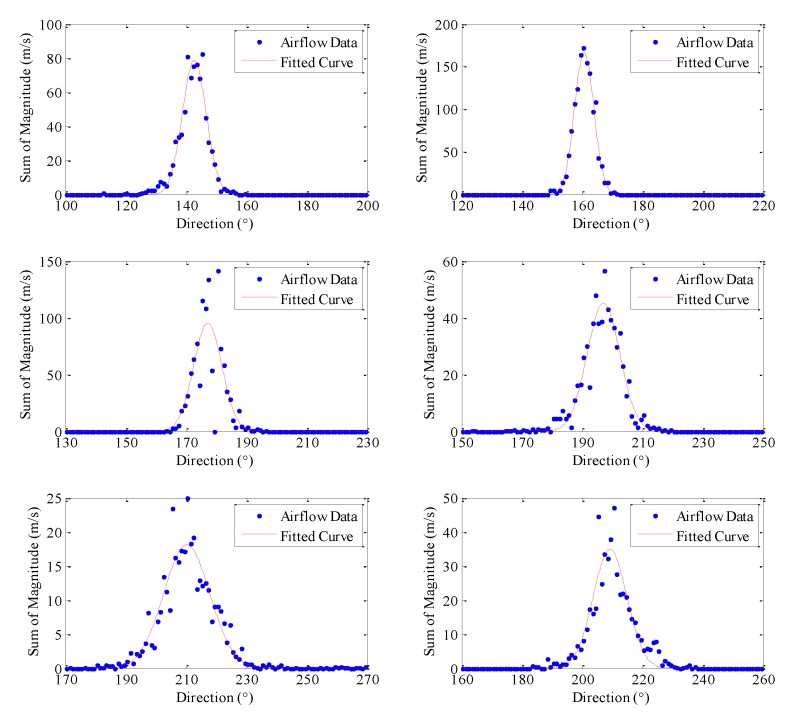
Distribution of airflow in the testbed.

The variation of the airflow was considered by dividing airflow vectors into 1° direction bins, and summing the magnitude of the airflow vectors in each bin. Interestingly, the distribution of airflow was observed to fit a one term Gaussian distribution for most positions in this experimental setup. The points where the distribution deviates from Gaussian distribution is when there are obstacle nearby or when the airspeed is very low. A graphical description of the airflow distribution is shown in Figure 13. The data collected in this testbed verify the assumption in odor dispersion models that the variations in air speed and direction fit to the Gaussian distribution.

#### 3.2.2. Gas Dispersion

**Figure 14:**
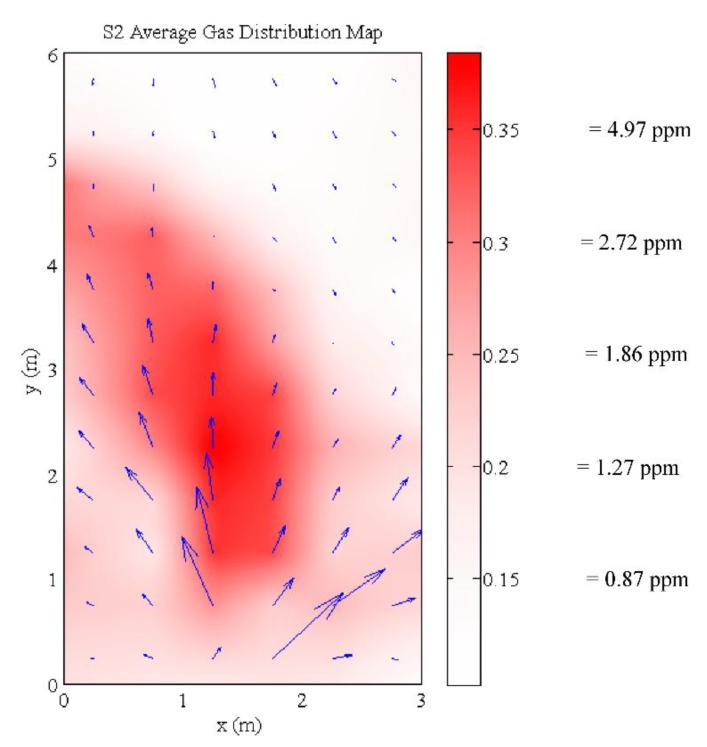
Average gas distribution map in testbed. The estimated ethanol concentration is described on the color scale.

The average dispersion in the testbed agrees with the measured airflow. The gas was distributed along the main airflow towards the bottom of Figure 14. The average concentration map agrees with most of the instantaneous concentration maps during the experiment. However, there are still differences in the instantaneous concentration maps as compared to the averaged concentration map.

**Figure 15:**
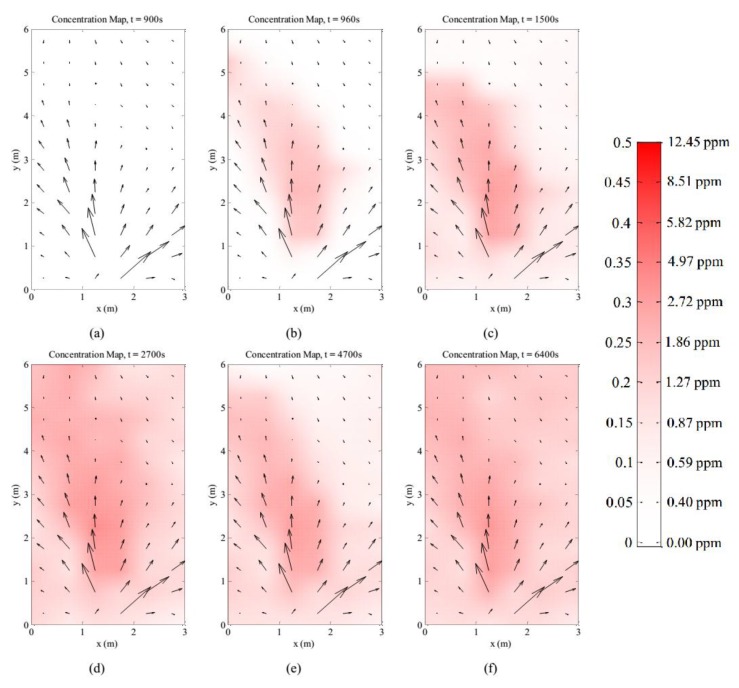
Snapshot of gas distribution map in testbed; (**a**) *t* = 900 s; (**b**) *t* = 960 s; (**c**) *t* = 1500 s; (**d**) *t* = 2700 s; (**e**) *t* = 4700 s and (**f**) *t* = 6400 s. The estimated ethanol concentration is described on the color scale.

The target gas was released into the testbed between *t* = 900 s to *t* = 8100 s. One minute after the gas was released, the detected plume resembles the average plume in terms of shape, although with lower concentrations. The plume keeps its shape and increases in concentration as shown in the concentration map at *t* = 1500 s. As the experiment continues, there appears to be a build-up of gas in the testbed. At *t* = 2700 s, although the main plume maintains the same shape, the surrounding area around the main plume records increased gas concentrations. The accumulation of the gas and the variability in the airflow contributes to the variability in the general shape of the gas plume as shown in the series of snapshots in Figure 15. It is worth noting that the area around the main plume accumulated significant levels of gas until the gas flow was stopped at *t* = 8100 s. After the gas flow was stopped, the concentration levels in the testbed decreases until it clears up at *t* = 11,700 s.

The instantaneous gas dispersion shows that on average, gas usually disperses in the general direction of the airflow through advection. However, the instantaneous gas dispersion can deviate far from the average gas dispersion map due to variation in airflow. The LGSA was able to capture the instantaneous dispersion of the gas in this experiment.

### 3.3. Gas Mapping Using LGSA

**Figure 16:**
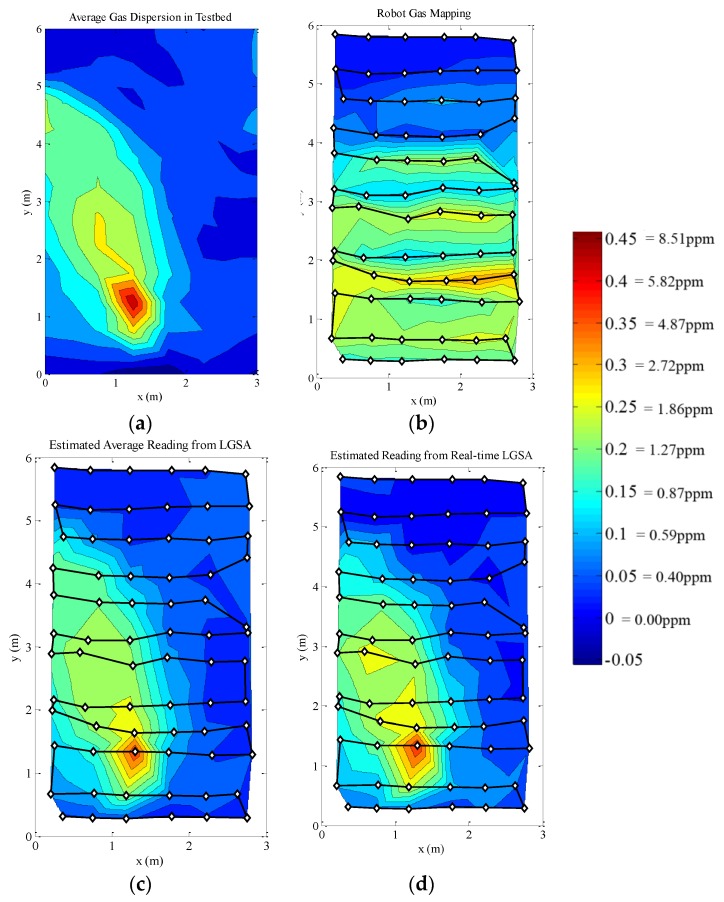
Gas distribution plots of constantly moving robot experiment. The robot’s movement path is denoted by the line and the dots indicate measurement points. The robot starts at the top right corner of the map. (**a**) The averaged LGSA reading for the duration of the experiment; (**b**) The measured gas concentration by the robot; (**c**) The ground truth robot reading estimated from the averaged LGSA measurement; (**d**) The ground truth robot reading estimated from the real-time LGSA measurements.

**Figure 17:**
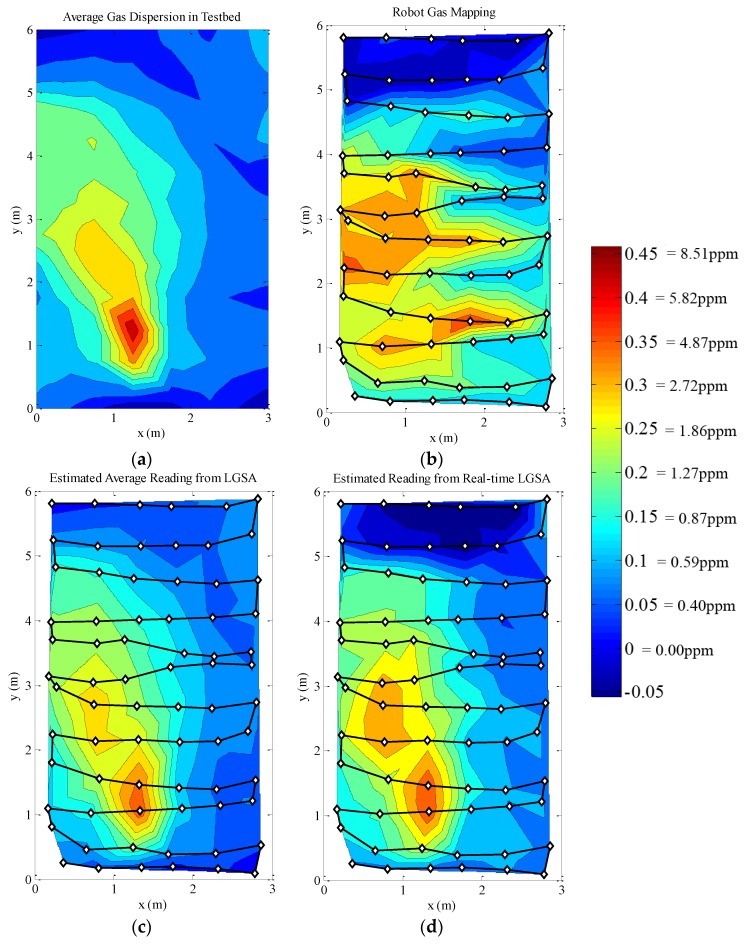
Gas distribution plots of stop–start robot experiment. The robot’s movement path is denoted by the line and the dots indicate measurement point. The robot starts at the top right corner of the map. (**a**) The averaged LGSA reading for the duration of the experiment; (**b**) The measured gas concentration by the robot; (**c**) The ground truth robot reading estimated from the averaged LGSA measurement; (**d**) The ground truth robot reading estimated from the real-time LGSA measurements.

Two sets of mobile olfaction experiments were conducted to gauge the performance of the LGSA by recreating the experiment as described in the previous section. The setup was chosen as it creates a relatively stable gas plume profile. The gas concentration measurements made by a robot is compared with the LGSA measurements. The robot used was a remote controlled National Instrument Robotics sbRIO Kit 2.0 equipped with a TGS2600 gas sensor. The gas sensor was placed at the highest point of the robot (approximately 19 cm from the ground) such that the gas sensor on the root is at the same height as the LGSA. Similarly, the LGSA was lowered to a height of 19 cm from the ground. The robot’s trajectory is depicted in Figure 16 and Figure 17.

The first gas distribution measurement was made by a constantly moving robot in the testbed area. In the second experiment, the robot stops for 5 s when taking gas concentration measurements. In both experiments, the LGSA was used to monitor the gas concentration in the testbed. In this section, the gas concentration maps for the robots are interpolated by triangulation. Contour maps are used to better depict the concentration levels and the shape of the gas plume.

As the gas sensor is slow, the gas concentration measurement made by the moving robot appears to be distorted compared to the averaged gas concentration. The gas sensor was unable to reach steady state as the robot was moving. The delay is obvious if the map is compared with Figure 16d, which is the expected gas measurement if the gas sensor response is ideally fast. Furthermore, the gas sensor signal increases and decreases slower and the peaks and troughs are detected at delayed positions; skewing the concentration maps. If the sensor was sufficiently fast and more data were sampled at each points, the gas concentration map produced would be similar to Figure 16c. To demonstrate this point, a second experiment was run with the robot stopping while making gas concentration measurements.

The stop–start measurement method produces a gas concentration map which is much closer to the time-averaged gas concentration map and reveals an obvious gas plume seen in Figure 17b. Even though the robot stops for 5 s while measuring the gas concentration, the robot was still unable to properly measure either the time-averaged or the instantaneous gas concentration levels. As such, similar distortions seen in the map created by a constantly moving robot is seen in the stop–start measurement; albeit less prominently. It is known that the robot affects the air movement in the testbed, and thus the gas dispersion in the testbed. However, no changes in the gas dispersion maps which can be attributed to the presence of the robot was observed. This may be due to the variability of the gas dispersion itself masking the effects of the robots presence. Furthermore, as the gas sensor on the robot and the LGSA is located higher than the bulk size of the robot, it is possible that the presence of the robot minimally affects the gas dispersion at the height of the gas sensors. The high net airflow at the gas sensors’ level may explain this phenomena. This experiment will not be pursued further in this document; however it has proven the need of ground truth knowledge to fully optimize and verify a mobile olfaction system.

## 4. Conclusions

In this work, a scalable system for mobile olfaction experimentation based on WSN backbone has been presented. Sensor calibration has verified that a large array of sensors can produce coherent sensor signals provided that the sensors are from the same production lot. The main component of the system, the LGSA, has been shown to be able to provide snapshots of the gas dispersion in the testbed which may aid to the design and test process of a mobile olfaction system. In addition, the collected results have shown the variability and the unpredictability in the gas dispersion, which information is lost in a time-averaged gas dispersion map. Furthermore, the system has demonstrated its usefulness in verifying the results of a gas distribution mapping experiment.

Further work includes improvements in gas distribution map plotting to create more accurate representation where various offline models have been proposed [32]. The gas sensor array may also be improved by increasing the number of sensor and integrating newer gas sensors which is faster, smaller in size and more energy efficient (e.g., MiCS-5526 or CCMOS CCS803 VOC sensor). It is envisaged that, with faster and more sensitive sensors, more variations in the gas plume may be observed thus providing better insight to the mobile olfaction problem. It is also possible to change the configuration of the LGSA to detect different types of gas by simply changing the gas sensors. However, as some types of gas sensors are slow and have relatively slower recovery times, the performance of the LGSA may differ depending on the types of gas sensor used.
